# Reliable enumeration of malaria parasites in thick blood films using digital image analysis

**DOI:** 10.1186/1475-2875-8-218

**Published:** 2009-09-23

**Authors:** John A Frean

**Affiliations:** 1 National Institute for Communicable Diseases, P/Bag X4, Sandringham 2131, Johannesburg, South Africa; 2 School of Pathology of the University of the Witwatersrand and the National Health Laboratory Service, Johannesburg, South Africa

## Abstract

**Background:**

Quantitation of malaria parasite density is an important component of laboratory diagnosis of malaria. Microscopy of Giemsa-stained thick blood films is the conventional method for parasite enumeration. Accurate and reproducible parasite counts are difficult to achieve, because of inherent technical limitations and human inconsistency. Inaccurate parasite density estimation may have adverse clinical and therapeutic implications for patients, and for endpoints of clinical trials of anti-malarial vaccines or drugs. Digital image analysis provides an opportunity to improve performance of parasite density quantitation.

**Methods:**

Accurate manual parasite counts were done on 497 images of a range of thick blood films with varying densities of malaria parasites, to establish a uniformly reliable standard against which to assess the digital technique. By utilizing descriptive statistical parameters of parasite size frequency distributions, particle counting algorithms of the digital image analysis programme were semi-automatically adapted to variations in parasite size, shape and staining characteristics, to produce optimum signal/noise ratios.

**Results:**

A reliable counting process was developed that requires no operator decisions that might bias the outcome. Digital counts were highly correlated with manual counts for medium to high parasite densities, and slightly less well correlated with conventional counts. At low densities (fewer than 6 parasites per analysed image) signal/noise ratios were compromised and correlation between digital and manual counts was poor. Conventional counts were consistently lower than both digital and manual counts.

**Conclusion:**

Using open-access software and avoiding custom programming or any special operator intervention, accurate digital counts were obtained, particularly at high parasite densities that are difficult to count conventionally. The technique is potentially useful for laboratories that routinely perform malaria parasite enumeration. The requirements of a digital microscope camera, personal computer and good quality staining of slides are potentially reasonably easy to meet.

## Background

Microscopic examination of Giemsa-stained blood films is widely relied upon for routine malaria diagnosis and particularly, for parasite density quantitation. Estimation of parasite burden in falciparum malaria is done for several reasons: as an indicator of risk of severe and complicated disease, especially in non-immune patients [1]; as a measure of response to treatment; as an aid to clinical decision-making about the likely cause of febrile illness in highly endemic areas [2]; and as an end-point in clinical trials of anti-malarial drugs or vaccines, at a pre-determined parasite density threshold [3].

It has been pointed out that there is a striking lack of evidence to support widely-held assumptions about the accuracy and consistency of malaria microscopy [2,4]. Studies have shown substantial intra- and inter-observer inconsistencies in density quantitation [2,5], and experience in proficiency testing of malaria microscopy in Africa supports this [6,7]. As counting parasites is tedious and tiring for microscopists, automation in the form of digital image analysis is an obvious potential solution [8]. Recent reports have described progress in applying this technology to thin film parasitaemia estimation [9-11]. However, despite some inherent limitations [4], thick blood films are more generally used for determining parasite density [12].

This contribution describes proof-of-principle of a simple, low-cost image analysis technique that is highly effective in enumerating moderate to high malaria parasite densities in thick blood films. Specific aims of this project were to develop a process to minimize user intervention, to avoid custom-written software or specialized hardware, and to thereby make the technique readily accessible to suitably-resourced laboratories.

## Methods

Thick blood films, Giemsa-stained to uniform standards [7] were obtained from our slide bank of malaria proficiency-testing specimens, or loaned from a similar collection (K. Lilley, Army Malaria Institute, Brisbane). Blood samples for malaria microscopy proficiency testing were collected and used with ethical approval of the Human Research Ethics Committee (Medical), University of the Witwatersrand, Johannesburg (protocol number M051126). Twenty films containing *Plasmodium falciparum *were selected to provide parasite densities ranging from 5,000 to 500,000 parasites/μl. These included eight re-sampled or duplicate slides to test reproducibility of methods both within and between films prepared from the same blood specimens. Parasite densities had been previously established by experienced microscopists using conventional counting methods [7,13]; namely, by counting parasites on thick films per 200 (or, in the case of very low densities, 500) leukocytes, multiplied by the patient's own leukocyte count, or if this was not available, a standard count of 8,000 leukocytes/μl. Specimens with very high counts (> 100 parasites per 100× objective field) were also counted on thin blood films as the proportion of infected erythrocytes multiplied by either the patient's red cell count, or, if this was not known, a standard red cell count (5 × 10^6 ^cells/μl).

Using a 50× objective in a conventional laboratory microscope (Olympus BX 41, Olympus Australia, Oakleigh, Victoria), sequential blood film images were captured by means of a Nikon DXM1200 digital camera [14] and Nikon ACT-1 software (Nikon Corporation, Tokyo, Japan) as uncompressed tagged image file format (TIFF) files at a resolution of 1,280 × 1,024 pixels. Apart from avoiding the irregular edges of the thick film and ensuring no overlapping of images, no special selection of captured fields was done. The number of leukocytes per image was recorded manually at the time of capture.

ImageJ (version 1.41)[15], an open-access Java-based image-processing programme, was used for image analysis. In essence, the programme segments or classifies particles to be counted on the basis of their relative density (darkness) compared with the background, via a thresholding process. Particle size (area) and degree of roundness are other classification variables. Fine morphological and differential staining characteristics of parasites are ignored. Therefore, non-parasite particles, that is, artifacts of various types, may also be segmented and are collectively termed noise (N). The target particles (malaria parasites) are the signal (S).

Precise enumeration of parasites per image (the 'gold standard' for this study), was done by manually counting parasites on the captured images (in total, about 98,000 parasites in 497 images from 20 specimens were counted). A 'Point Picker' plugin [16] that digitally tags each counted parasite and records its coordinates for future reference, was used to facilitate manual counting. Using the particle analysis commands of ImageJ, parasites were then counted digitally on the same images. Between 20 and 30 (mean, 25) images per slide were analysed simultaneously in a stack; the amount of virtual memory available to the image analysis software constrains stack size. Three hundred images, representing 12 different slides (calibrators), were used in the calibration experiments described below; 197 new images from eight re-sampled or duplicate slides were used to validate the findings and assess reproducibility.

Statistical evaluations were done using Statistica 8.0 (StatSoft, Tulsa, OK). Because of non-normal distributions of data sets and small sample sizes (n < 30), non-parametric tests were used. Statistical evaluation at individual slide level was by signed rank tests that compared numerical results of manual and digital counting methods, together with the rank order correlation coefficient (R) as a measure of reliability of digital counts. Non-parametric ANOVA was used to compare collective counts by the three methods (conventional, manual, and digital). World Health Organization (WHO) criteria for evaluating accuracy of counts done by working microscopists, expressed as the percentage absolute discrepancy between experimental and reference counts, were used in a less stringent but practical comparison system [12].

## Results

During initial experiments to obtain a standard particle analysis algorithm applicable to thick film images, it was apparent that more user intervention, in terms of adjusting various algorithm variables, was required for films with lower parasite densities and/or later-stage trophozoites. Ultimately, a single adjustment factor was identified that would accommodate nearly all densities and sizes of parasites. This factor, designated RN, is the radius of the area used in the 'Remove outliers' command, 'Noise' submenu, 'Process' menu, of ImageJ. The command replaces a pixel by the median of the pixels in the surrounding area if it deviates from the median by more than a certain threshold value; the effect is to smooth irregular shapes and reduce non-specific noise, with increasing rigor as RN increases. Above a certain value of RN, signal is also removed. The effect of this on counts is variable and depends on the relative proportions of signal and noise.

**Figure 1:**
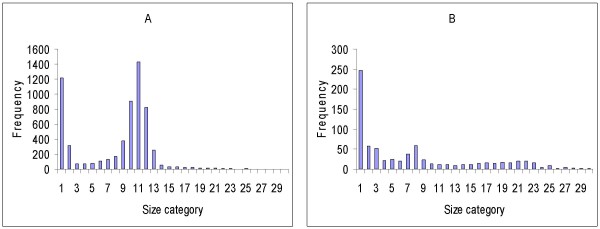
**Examples of particle size frequency distributions**. Results of preliminary process (Macro 1) on image sets of 2 slides. A. BF3A: particle count = 7305; kurtosis (K) = 5.81; skewness (Sk) = 2.49; K/Sk = 2.33; RN = 5.2. B. BF8A: particle count = 772; kurtosis (K) = 22.8; skewness (Sk) = 4.56; K/Sk = 5; RN = 7.02.

**Figure 2:**
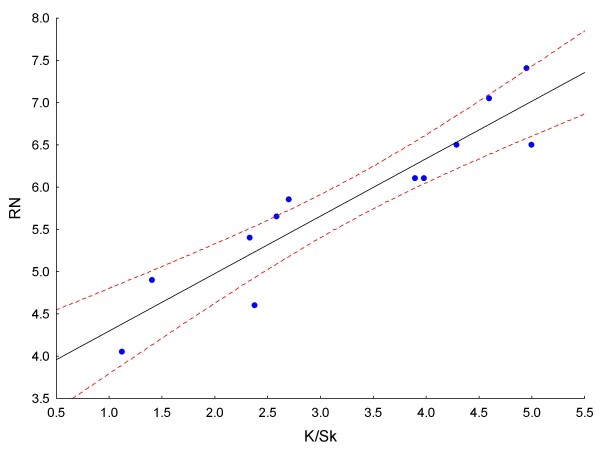
**Linear regression of K/Sk on RN**. Regression line equation: y = 0.6795x + 3.6188; R = 0.93; dashed lines are 95% confidence limits.

**Figure 3:**
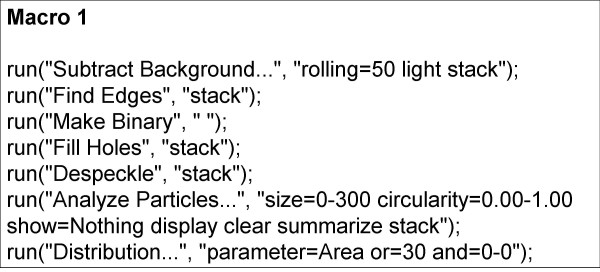
**Macro 1**. This preliminary process determines the K, Sk, and resultant RN values for each image stack.

**Figure 4:**
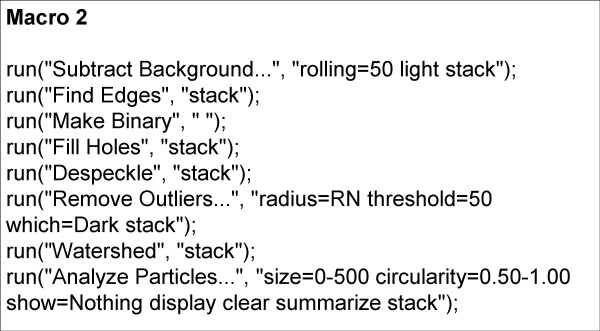
**Macro 2**. This algorithm produces the final parasite count for each image stack.

Iterations with various RN values showed that for each specimen there was a small range of RN that produced an optimum signal/noise (S/N) ratio and, therefore, an optimum particle count, compared to the known (manual) count. Experiments with various measures of dispersion or scatter of particle size about the mean ultimately showed that for every specimen analysed, except for those with very low counts (fewer than about 140 parasites counted per 25-image stack), the mean RN value was directly proportional to both the skewness (Sk) and the kurtosis (K) of the particle size distribution. Figures 1A and 1B show how K and Sk vary between particle size frequency distributions of different specimens. The best correlation obtained (R = 0.93) was between the kurtosis/skewness (K/Sk) ratio and mean RN (Figure 2); 300 images, representing 12 different smears (calibrators), were used to generate the curve. This suggested a method to automatically determine the best RN value for each slide and minimize user intervention in obtaining the optimal count. Essentially, preliminary processing and particle counting using Macro 1 (Figure 3) on the stack of thick film images captured from each slide yielded a frequency distribution of particle size. These data were copied (by ImageJ command) to an Excel spreadsheet, and Sk and K were elicited via the spreadsheet's descriptive statistics tool. Using the formula of the regression line (RN = 0.6795 × K/Sk + 3.6188), a value for RN was obtained. This was applied in Macro 2 (Figure 4) to obtain the parasite count for the stacked images. This count was combined with the corresponding manual leukocyte count (as in conventional quantitation, described above) to produce the final result as number of parasites per μl.

**Figure 5:**
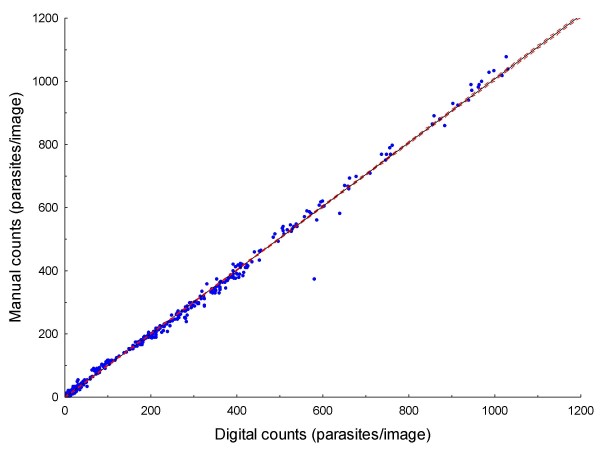
**Linear regression of digital counts on manual counts of 497 images**. R = 0.99; dashed lines are 95% confidence limits.

**Figure 6:**
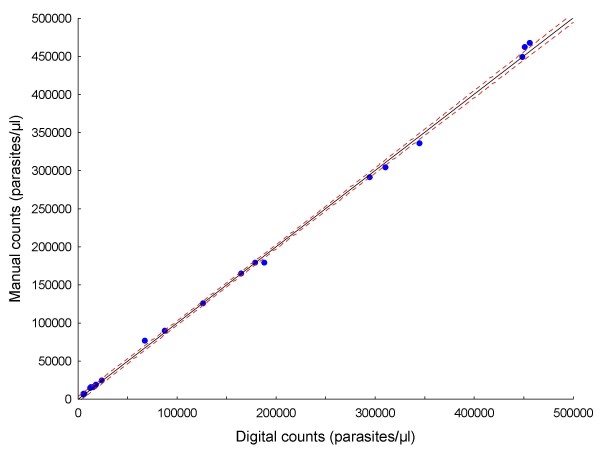
**Linear regression of aggregated digital counts on manual counts of 20 slides**. R = 0.99; dashed lines are 95% confidence limits.

**Figure 7:**
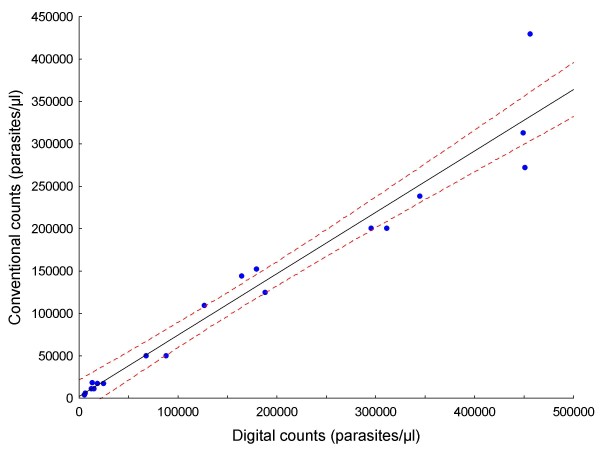
**Linear regression of aggregated digital counts on conventional counts of 20 slides**. R = 0.97; dashed lines are 95% confidence limits.

**Figure 8:**
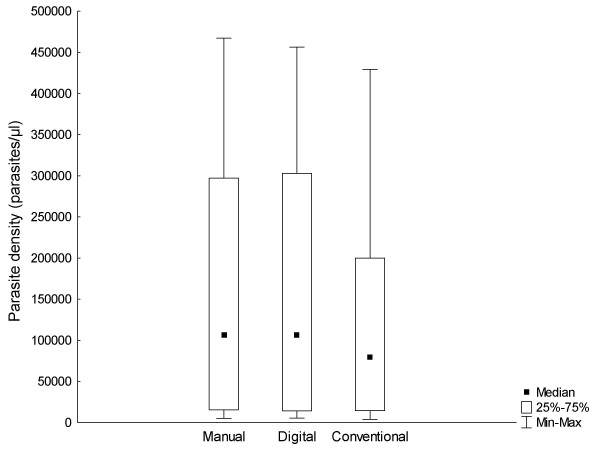
**Box plots of aggregated manual, digital and conventional counts**.

Original data for all analysed images are provided in Additional file 1: Count data.xls. Results are summarized in Tables 1 and 2. Figure 5 is the plot of the linear regression of digital counts on manual counts for all of the images (n = 497), showing a strong correlation between them (R = 0.99) and narrow 95% confidence intervals. Comparing digital counts with manual counts (the gold standard), the mean absolute error per slide was 4.74% (range, 0.06% - 15.99%; Table 1). Aggregated counts per slide (expressed as parasites per μl) generated by the digital image analysis process correlated well both with those derived manually in this study (R = 0.99; Figure 6), and by conventional methods (R = 0.97; Figure 7). The aggregated parasite densities (in parasites/μl) per slide produced by the three methods differed significantly (Friedman ANOVA, p < 0.001) and inspection of the data showed that with one exception (BF10A), conventional counts were lower than corresponding digital and manual counts (Table 1; Figure 8). The absolute mean percentage discrepancy between manual and conventional counts was 23.7% (range 7.4 - 43.7%). Some slides' sets of analysed images showed significant differences between manual and digital counts (Wilcoxon signed rank test, Table 2); however, the percentage differences in all cases were well below the 25% acceptable discrepancy limit that WHO recommends [12]. Correlation (Spearman rank order) between manual and digital counts across the same sets of images showed some variation, with lower counts tending to produce lower correlation coefficients (Table 2). Only one slide (AMI26) had a non-significant correlation (R = 0.24; p = 0.08). Although the Wilcoxon test indicated no significant difference between manual and digital counts for this slide (p = 0.53), and the percentage difference was within acceptable limits (10.24%; Table 1), the low correlation coefficient indicates substantial misclassification of particles, and a suboptimal S/N ratio. Misclassification is associated with low parasite densities because for the equivalent amount of noise removed, the simultaneous removal of parasites by the noise-reduction algorithm has a relatively greater effect on S/N ratio at low parasite densities, compared to high ones.

**Table 1:** Comparison of manual, digital, and conventional parasite density estimations

**Slide**	**Number of parasites counted per slide**		**WC**	**WCC**	**Parasite density, as parasites/μl**		
				
	**Manual counts**	**Digital counts**			**Manual counts**	**Digital counts**	**Conventional counts**
BF3A	5022	5071	323	8050	125161	126383	109725
BF5A	4516	4540	162	6400	178410	179358	152178
BF6A	10686	10438	118	5100	461853	451134	271600
BF7A	9373	9515	316	9800	290682	295085	200083
BF8A	344	289	125	2510	6908	5803	5834
BF9A	634	619	202	5020	15756	15383	11359
BF10A	1003	870	353	5400	15343	13309	18638
BF13A	2446	2158	290	9070	76501	67493	50110
BF18A	22477	21956	534	11100	467219	456389	429400
AMI3a	424	438	142	8000	23887	24676	17500
AMI26	127	140	200	8000	5080	5600	3975
AMI32	5819	6149	261	8000	178360	188475	125000
BF7Ab	8059	8251	260	9800	303762	310999	200083
BF8Ab	291	277	109	2510	6701	6379	5834
BF9Ab	369	333	132	5020	14033	12664	11359
BF13Ab	1373	1356	140	9070	88951	87849	50110
BF15A	3904	3914	152	6400	164379	164800	144050
BF16A	14265	14273	162	5100	449083	449335	313062
BF17A	6807	6999	199	9800	335219	344674	238467
AMI3b	281	272	119	8000	18891	18286	17500
							
Total	98220	97858			3226178	3224075	2375867
Mean			215				

Data are kurtosis/skewness (K/Sk) and resultant factor RN, used to set digital particle counting algorithm for each slide; number of particles per slide counted manually and digitally, and compared by absolute % discrepancy, rank correlation, and signed rank test.

**Table 2:** Comparison of manual and digital counts

**Slide**	**K/Sk**	**RN**	**Number of parasites counted per slide**		**% Discrepancy**	**Spearman correlation R; p**	**Wilcoxon test p**
						
			**Manual counts**	**Digital counts**			
BF3A	2.33	5.20	5022	5071	0.98	0.99; < 0.05	0.15
BF5A	2.59	5.38	4516	4540	0.53	0.98; < 0.05	0.36
BF6A	1.13	4.38	10686	10438	2.32	0.96; < 0.05	< 0.01
BF7A	2.70	5.45	9373	9515	1.51	0.94; < 0.05	0.03
BF8A	5.00	7.02	344	289	15.99	0.56; < 0.05	0.01
BF9A	4.29	6.53	634	619	2.37	0.76; < 0.05	0.40
BF10A	3.98	6.32	1003	870	13.26	0.62; < 0.05	< 0.01
BF13A	3.90	6.27	2446	2158	11.77	0.91; < 0.05	< 0.01
BF18A	2.38	5.24	22477	21956	2.32	0.98; < 0.05	< 0.01
AMI3a	1.41	4.57	424	438	3.30	0.61; < 0.05	0.53
AMI26	4.60	6.74	127	140	10.24	0.24; 0.08	0.53
AMI32	4.95	6.98	5819	6149	5.67	0.91; < 0.05	< 0.01
BF7Ab	2.31	5.19	8059	8251	2.38	0.99; < 0.05	< 0.01
BF8Ab	4.63	6.76	291	277	4.81	0.69; < 0.05	0.08
BF9Ab	4.27	6.52	369	333	9.76	0.85; < 0.05	0.03
BF13Ab	3.18	5.78	1373	1356	1.24	0.88; < 0.05	0.14
BF15A	2.55	5.35	3904	3914	0.26	0.99; < 0.05	0.63
BF16A	1.71	4.78	14265	14273	0.06	0.94; < 0.05	0.03
BF17A	2.21	5.12	6807	6999	2.82	0.86; < 0.05	< 0.01
AMI3b	1.30	4.50	281	272	3.20	0.97; < 0.05	0.33
							
Total			98220	97858			
Mean					4.74		

Data are kurtosis/skewness (K/Sk) and resultant factor RN, used to set digital particle counting algorithm for each slide; number of particles per slide counted manually and digitally, and compared by absolute % discrepancy, rank correlation, and signed rank test.

Reproducibility was tested by comparing digital counts done on re-sampled (same slide, different set of images; n = 5) and duplicate (same original blood specimen, different slide; n = 3) slides (Table 3). The mean absolute percentage discrepancy between counts was 14.3% (range, 0.4 - 30.2%) and original counts and recounts did not differ significantly overall (p = 0.58, Wilcoxon signed rank test). For the corresponding manual counts, results were similar (mean absolute discrepancy 10.2%; range, 2.8 - 20.9%; p = 1). (Equivalent re-sampling data were only available for 3 of the conventional counts and they were therefore not analysed.)

**Table 3:** Comparison of digital parasite density estimations on pairs of duplicate or re-sampled* slides

**Slide pairs**	**Parasite density, as parasites/μl**		**% discrepancy**
		
	**Slide 1**	**Slide 2**	
BF5A/BF15A	179358	164800	8.1
BF6A/BF16A	451134	449335	0.4
BF7A/BF17A	295085	344674	16.8
*BF7A/BF7Ab	295085	310999	5.4
*BF8A/BF8Ab	5803	6379	9.9
*BF9A/BF9Ab	15383	12664	17.7
*BF13A/BF13Ab	67493	87849	30.2
*AMI3a/AMI3b	24676	18286	25.9
			
Mean			14.3

## Discussion

Errors in parasite density estimation by conventional microscopy are common, and apart from possibly deleteriously influencing the management of individual patients, have the potential to produce major consequences for clinical efficacy trials of malaria vaccines or prophylactic drugs [6,17]. Utility of automated digital particle analysis for enumerating parasites has previously been limited by the variability of size, shape, and staining characteristics of asexual malaria parasites on conventional stained thick blood films. In this study, highly accurate manual counts of a range of parasite densities made it possible to experiment extensively with digital counting methods, and to critically evaluate particle analysis algorithms. In addition, the accuracy of conventional counting methods applied to the same slides was assessed.

With ordinary image analysis particle counting, it is straightforward to adjust algorithm variables to achieve highly accurate counts on individual slides when the target (manual) count is known. However, this is clearly not relevant to routine parasite density determination where a set target is absent, and adjusting algorithm variables subjectively introduces unknown biases into counts. Variation in parasite size and shape preclude use of a universal algorithm, with or without additional manual adjustment. The method described here circumvents this problem by providing semi-automatic adjustment of the most important variable controlling the S/N ratio, based on certain frequency distribution parameters (kurtosis and skewness) of the particles being analysed. It can be seen that there are no decisions required from the user that might introduce subjectivity or bias.

Generally, digital counts correlated well with both manual and conventional counts (Figures 6 and 7), but conventional counts were significantly lower than digital and manual counts. In conventional counting of relative numbers of parasites and leukocytes, human operator biases, which are absent in the digital and manual counts described here, presumably account for this tendency to underestimate parasite densities. The method of digital counting described here essentially solves the problem of counting parasites at medium to high densities, but in images with low absolute numbers of parasites (< 140 parasites per 25 images), there is evidence that lower S/N ratios, because of misclassification, constrain the accuracy of counts. It may be possible to predict when digital counts are likely to be unreliable; in this data set it appears that when the quotient of the digital parasite count and RN is less than 20, a suboptimal S/N ratio is likely, despite the fact that the total count may be within acceptable limits. Further experiments will be required to verify this finding. Generally, lower densities are technically easier to count conventionally than high ones and, therefore, this restriction is not a practical problem (notwithstanding error due to sample distribution effects at low densities).

With regard to reproducibility of digital counts within and between films made from the same blood specimens, the data set is small, but simultaneous comparison with manual counts suggests that differences in digital counts in this subset of slides were mainly due to real inter-sample variation, with only a small contribution from counting errors.

Cost and availability of equipment may constrain application of image analysis; a digital camera-equipped microscope and a computer are required. Another limitation of the technique is that stain and other artifacts may contribute to unwanted noise. It follows that good-quality staining with minimal residual stain precipitate is necessary for optimum results. Visual inspection of smears during image capture should alert the microscopist to significant numbers of Howell-Jolly bodies, yeasts, or other particles that may also occasionally contribute to noise. Dim images tend to be noisy; it is important to optimize the brightness and contrast of captured images for automatic thresholding by the image analysis software. Another constraint is the need to capture substantial numbers of digital images of the specimens, which is not difficult but is time-consuming, taking up to an hour to carefully capture 25 images. Computer-controlled motorized microscope stages and automatic focusing are solutions to this problem that are already available from some manufacturers, but would add to costs. In contrast, the subsequent digital counting process is fast, requiring about 5 to 10 minutes per specimen to complete.

## Conclusion

This proof-of-principle study has shown that it is possible to achieve high standards of accuracy and reproducibility of thick film malaria parasite counts by digital image analysis. Digital counts were generally well within the acceptable limits of accuracy recommended by the WHO [12]. High quality free software and semi-automation of the counting process make this technique potentially widely accessible to many diagnostic laboratories. Further evaluation of the method using a larger number of malaria blood films and different digital camera systems, is the next objective. The principle may suggest a general solution for automated biological particle counting, with minimal operator input required, when some variation in size and shape of the target is present.

## Competing interests

The author declares that he has no competing interests.

## Authors' contributions

JF conceived, designed, and performed the studies described here whilst on sabbatical leave at the Australian Army Malaria Institute (AMI), Brisbane, November 2008 - May 2009.

## Supplementary Material

Additional file 1 **Manual and digital counts of all images (n = 497)**. Excel spreadsheet; data are grouped by slide identifier; manual and digital counts for each image, and the aggregated count for each slide, are provided. Click here for file
